# Endothelial colony forming cell administration promotes neurovascular unit development in growth restricted and appropriately grown fetal lambs

**DOI:** 10.1186/s13287-023-03249-z

**Published:** 2023-02-14

**Authors:** Alexander Bell, Ashalyn P. Watt, Ingrid Dudink, Yen Pham, Amy E. Sutherland, Beth J. Allison, Courtney A. McDonald, Margie Castillo-Melendez, Graham Jenkin, Atul Malhotra, Suzanne L. Miller, Tamara Yawno

**Affiliations:** 1 The Ritchie Centre, Hudson Institute of Medical Research, Melbourne, Australia; 2 Department of Obstetrics and Gynaecology, Monash University, Melbourne, Australia; 3 Department of Paediatrics, Monash University, 246 Clayton Road, Clayton, Melbourne, VIC 3168 Australia; 4 Monash Newborn, Monash Children’s Hospital, Melbourne, Australia

**Keywords:** Brain injury, Blood brain barrier, Cord blood, Endothelial progenitor cells, ECFC, FGR, Repair, Stem cells

## Abstract

**Background:**

Fetal growth restriction (FGR) is associated with deficits in the developing brain, including neurovascular unit (NVU) dysfunction. Endothelial colony forming cells (ECFC) can mediate improved vascular stability, and have demonstrated potential to enhance vascular development and protection. This investigation examined whether ECFCs from human umbilical cord blood (UCB) enhanced NVU development in FGR and appropriate for gestational age (AGA) fetal sheep.

**Methods:**

Twin-bearing ewes had surgery performed at 88–90 days’ gestation, inducing FGR in one fetus. At 113 days, ECFCs (1 × 10^7^ cells) cultured from human UCB were administered intravenously to fetal sheep in utero. At 127 days, ewes and their fetuses were euthanised, fetal brains collected, and NVU components analysed by immunohistochemistry.

**Results:**

Twenty-four fetal lambs, arranged in four groups: AGA (n = 7), FGR (n = 5), AGA + ECFC (n = 6), and FGR + ECFC (n = 6), were included in analyses. FGR resulted in lower body weight than AGA (P = 0.002) with higher brain/body weight ratio (P = 0.003). ECFC treatment was associated with increased vascular density throughout the brain in both AGA + ECFC and FGR + ECFC groups, as well as increased vascular–astrocyte coverage and VEGF expression in the cortex (P = 0.003, P = 0.0006, respectively) and in the subcortical white matter (P = 0.01, P = 0.0002, respectively) when compared with the untreated groups.

**Conclusions:**

ECFC administration enhanced development of NVU components in both the AGA and FGR fetal brain. Further investigation is required to assess how to optimise the enhanced angiogenic capabilities of ECFCs to provide a therapeutic strategy to protect the developing NVU against vulnerabilities associated with FGR.

## Background

Fetal growth restriction (FGR) is defined by the failure of a fetus to achieve their genetic growth potential and is frequently cited as among the most common complications of pregnancy. Estimates of FGR prevalence have ranged from 5 to 15% of pregnancies [1], though the historical lack of a consensus definition and underdiagnosis of the condition make the true figure difficult to accurately identify [2]. Recent data, using the 2016 consensus definition describing FGR as an infant who is < 3rd percentile estimated weight for gestation or < 10th percentile with evidence of uteroplacental dysfunction [3], indicated that 6.9% of pregnancies met this criteria [4]. This definition reflects that FGR most commonly arises due to an inadequate supply of oxygen and nutrients to the fetus caused by placental insufficiency [5]. Fetal adaptations to reduced placental supply preferentially redirect blood flow to the brain [6, 7]; however, the trajectory of brain development remains altered in FGR [8].

FGR is associated with a broad range of structural deficits in the brain across both grey and white matter regions that underpin functional impacts affecting motor, cognitive, and behavioural performance in early life and beyond [9]. We have recently highlighted the potential role of neurovascular unit (NVU) dysfunction as a cause and contributor to neurodevelopmental impairments that are present in FGR infants [6]. The NVU is a collection of closely associated cellular and extracellular structures, including vascular cells, glial cells, and neurons, which operate collectively to match vascular function with neuronal metabolism [10]. FGR is linked to impairments in the developing NVU including reduced vascular glial coverage and endothelial proliferation, as well as potentially devastating vascular sequelae such as intraventricular haemorrhage [11–13], with studies suggesting treatments targeting these impairments may ameliorate injury in the brain [14, 15].

Endothelial progenitor cells (EPC) represent a broad category of cells that can be further divided into early EPCs and late EPCs (or endothelial colony forming cells (ECFC)), depending on their stage of development. Studies investigating EPCs as a potential therapeutic treatment in neurovascular disease show promising results [16–18]. Investigations using adult animal models of ischaemic and haemorrhagic brain injury have demonstrated both protective and regenerative benefits with EPC treatment, though these have largely used early EPCs derived from bone marrow. Benefits include increased vascular proliferation, increased proangiogenic signalling and barrier protein expression, reductions in infarct sizes and cortical atrophy, and enhanced neurobehavioural outcomes [16–18]. Such models have also demonstrated a capacity for EPC treatments to ameliorate deficits in blood brain barrier (BBB) function resulting from a variety of neurovascular insults [19–21]. Though both early EPCs and ECFCs express proangiogenic function, ECFCs have a higher proliferation rate and survival, and appear to best exhibit the function and phenotype expected in progenitor cells of the endothelial lineage [22–24]. ECFCs are particularly effective at stimulating neovascularisation and promoting regeneration of damaged tissues, through both direct engraftment into vessels and paracrine signalling using growth factors and cytokines acting on NVU components [25, 26]. This signalling enables ECFCs to act not only to stimulate angiogenesis, but to enhance the stability and repair of other NVU components such as the glial cells that experience reduced vascular coverage in FGR [27, 28]. Given that FGR is linked to neuroinflammation exacerbated by associated dysfunction in developing NVU structures and functions, such as BBB regulation [29], this raises the possibility that ECFCs could be used as a targeted neuroprotective therapy in FGR. To date, however, studies of therapeutic administration of EPCs or ECFCs in perinatal brain injury are scarce. Fetal and neonatal animal models have, however, been used to demonstrate the utility of umbilical cord blood (UCB) containing these cells for the treatment of acute hypoxic-ischaemic brain injury [30–33]. Our group has previously showed that unlike other populations of stem and progenitor cells within UCB, early EPCs are capable of modifying peripheral immune response towards a more protective environment, and these cells demonstrate neuroprotective effects [30]. Findings from these preclinical studies have led to the suggestion that administration of EPCs or ECFCs isolated from UCB may provide superior benefits for perinatal brain injury, compared with UCB mononuclear cells [30]. Though interactions between the immune system and ECFCs remain unclear, evidence that these cells are tolerated against allogeneic immune responses [34–36] provides further encouragement that these cells may provide an ‘off-the-shelf’ treatment option.

ECFCs in UCB are more concentrated and express greater functional potential than those isolated from other sources, such as peripheral blood [37]. UCB concentration of early EPCs is reduced in human pregnancies compromised by FGR compared to normal pregnancy [38], with the differentiation of these cells into ECFCs also impaired [39]. Given the role that EPCs and ECFCs play in vascular homeostasis, this suggests that low circulating fetal levels of these cells may play a role in the altered fetal brain development observed in FGR [8, 9]. Here, we proposed that therapeutic administration of ECFCs to FGR offspring could provide a novel treatment targeted at preventing NVU dysfunction and brain injury in the developing brain, especially in cases of FGR. Accordingly, this proof-of-concept study evaluated whether exogenous ECFC administration to growth restricted fetuses could reduce NVU damage/abnormalities and promote vascular protection.

## Methods

### Ethics approval

Experiments complied with the National Health and Medical Research Council (NHMRC) of Australia guidelines for the care and use of animals for scientific purposes and were approved by the Monash Medical Centre Animal Ethics Committee (MMCA2019/04). Ethics approval to obtain UCB for this study was obtained from Monash Health Human Ethics Committee (HREC Approval 12387B Ver 18).

### Surgical preparation and tissue collection

Date-mated pregnant Border Leicester-Merino crossbred ewes with known gestational age were obtained from the Monash Animal Research Platform. Twenty-four fetal lambs collected from thirteen twin-bearing ewes were randomised to four different groups: Appropriate for Gestational Age (AGA) (n = 7), FGR (n = 5), AGA + ECFC (n = 6), and FGR + ECFC (n = 6). Animals were housed in approved facilities and continuously monitored for the duration of the investigation. Sterile surgery was performed on twin-bearing Border–Leicester crossbred ewes at a known gestational age of 88–90 days (0.6 gestation; term is ~ 150 days), with the surgical procedure to induce FGR performed as previously described [14, 15, 40]. This gestational age was selected to represent early-onset FGR, which is associated with more widespread cerebral damage than late-onset FGR [41]. With the ewe under general anaesthetic (isoflurane, 1.5–2.5% in O_2_; Bomac Animal Health, Australia) each fetus was accessed via an abdominal and uterine incision. A single catheter (inner diameter [ID] 0.8 mm, outer diameter [OD] 1.5 mm) was placed into the fetal jugular vein in the treatment cohort only. For all cohorts, we performed single umbilical artery ligation on one fetus by placing two tight silk ligatures around one umbilical artery, approximately 3–4 cm from the fetal abdomen; in the other fetus (control/ AGA), the umbilical cord was manipulated but ligation was not performed. Additional catheters were placed into the maternal jugular vein (ID 1.5 mm, OD 2.7 mm) and the fetal amniotic space (ID 1.5 mm, OD 2.7 mm), before the fetuses were returned to the uterus, and the uterus and abdomen repaired in layers. Anaesthetic was withdrawn from the ewe and she was returned to a pen for recovery. Antibiotics were administered to both the ewe (engemycin, 5 ml, ampicillin, 1 g) and each of the fetuses (ampicillin, 500 mg) on each of the first three days following surgery. Animals were monitored daily, with fetuses in the ECFC groups receiving treatment of 1 × 10^7^ ECFCs intravenously in 2 ml phosphate buffered saline (PBS) at a gestational age of 113 days.

At approximately 127-day gestation, ewes and fetuses were euthanised by maternal overdose of pentobarbitone sodium (100 mg/kg, i.v.; Virbac Pty Ltd Australia), before fetuses were removed from the uterus and weighed. The fetal brain was removed, weighed, and cut along the sagittal plane to separate the two hemispheres, with the left hemisphere snap frozen into major anatomical regions and the right hemisphere cut along the coronal plane into sections of approximately 5 mm thickness. These coronal sections were fixed by immersion in 10% buffered formalin for three days before being embedded in paraffin. We used coronal sections of 10 μm thickness cut from these blocks by microtome at the level of the lateral ventricle and loaded onto Superfrost Plus slides (Thermo Fisher Scientific, MA, USA) for analysis.

### Human ECFC culture and preparation

UCB was collected at healthy term elective caesarean section delivery after consent was obtained from seven donors. Following delivery of the placenta, UCB was collected into bags containing CPD anticoagulant (Macopharma, Australia). The mononuclear cells (MNC) were isolated as previously described [42, 43], using 50 ml SepMate tubes (Stemcell Technologies, Canada) containing 13 ml Ficoll-Paque Plus (GE Healthcare, Sweden).

To culture ECFCs from UCB, 6-well tissue culture plates were coated with fibronectin (2 µg/cm^2^). UCB MNC were plated at a density of approximately 5 × 10^6^/well in ECFC culture media containing endothelial basal media (EBM-2, Lonza, MD, USA) supplemented with 20% fetal bovine serum (FBS), 2 µg/ml ascorbic acid (Sigma-Aldrich, MO, USA), 100 U/ml heparin (Sigma-Aldrich, MO, USA), 20 ng/ml insulin-like growth factor-1 (Miltenyi Biotec, Germany), 25 ng/ml vascular endothelial growth factor (Miltenyi Biotec, Germany), 10 ng/ml fibroblast growth factor-2 (Miltneyi Biotec, Germany), 10 ng/ml epidermal growth factor (Miltenyi Biotec, Germany), 100 ng/ml hydrocortisone (Sigma-Aldrich, MO, USA), and 1 × Penicillin–Streptomycin (Thermo Fisher Scientific, NY, USA), and placed in a 37 °C, 5% CO_2_ incubator. After four days of culture, a 50% media change was done and repeated every second day. Plates were monitored for colony formation and once observed were allowed to proliferate for approximately 7 days before being harvested. Colonies were harvested using cloning rings and accutase solution (Thermo Fisher Scientific, CA, USA) to detach the cells from the plates. Colonies were then replated in 25 cm^2^ tissue culture flasks coated with 2 µg/cm^2^ fibronectin and cultured in ECFC culture media with the exception that the FBS was reduced to 10% hereafter. At near confluence (80%) cells were subcultured into 75 cm^2^ tissue culture flasks coated with fibronectin, ECFCs were cultured until passages 3–5 for use for subsequent experiments. ECFCs were cryopreserved at a density of 5 × 10^6^ cells/ml in freeze medium containing 90% FBS and 10% dimethyl sulfoxide (Sigma-Aldrich, MO, USA).

To prepare ECFCs for administration, cryovials were removed from liquid nitrogen storage and thawed in a water bath at 37 °C until only a small ice crystal remained. Thawed cells (2 × 10^7^) were transferred into a 15 ml falcon tube with the remainder of the tube filled with Dulbecco's Modified Eagle Medium (Life Technologies, Australia), and the tube centrifuged at 300×g for 5 min, 4 °C to pellet the cells. Cells were washed with 1 × PBS and pelleted by centrifugation at 300×g for 5 min, 4 °C. Pelleted cells were resuspend in Diluent C (Sigma-Aldrich, MO, USA) and fluorescently labelled with PKH26 ethanolic dye solution (Sigma-Aldrich, MO, USA) according to manufacturer’s instructions, so that it could be determined via fluorescence microscopy whether ECFCs administered to fetal lambs had integrated into the NVU. Cells were prepared for administration by resuspending in 4 ml 1 × PBS then drawing up 2 ml of the cell solution into each of two separate 3-ml syringes. Cells were administered to the fetal sheep via the 3-ml syringe, with each fetus receiving 1 × 10^7^ cells into the fetal jugular vein catheter over a 2 min slow infusion, followed by a 10 ml saline flush delivered as a bolus.

### Tube formation assay

To confirm ECFC phenotype, cells were assessed for their ability to form tubes using in vitro tube formation assay. For the tube formation assay, 200 µl of growth factor reduced Matrigel (BD) was added to the bottom of a 24 well plate and allowed to set for 1 h in a 37 °C incubator. ECFCs (1 × 10^5^) were plated in ECFC culture media and incubated for 24 h. Images were taken on an Olympus CX53 microscope.

### acLDL uptake assay

Another characteristic used to confirm ECFC phenotype is uptake of acetylated low-density lipoproteins (acLDL) [44]. ECFCs (1 × 10^4^) were plated in Nunc 4 well Lab TEK II chamber slides (Sigma-Aldrich, NY, USA) in ECFC culture media overnight. The next day media was removed and replaced with serum-free culture media for 6 h before staining. 5 µg/ml of acLDL-DiL (Thermo Fisher Scientific) was added to the well for 4 h, and then, cells were washed with PBS and counterstained with 1 µg/ml Hoechst 33,342 (Thermo Fisher Scientific, OR, USA) for 3 min. For imaging, DAKO fluorescence mounting medium (Agilent, CA, USA) was added to the slide and coverslip applied.

### Flow cytometry

Cultured ECFCs were assessed for their surface expression of common ECFC markers. Flow cytometric analysis for surface markers was performed using the following antibodies: CD14 FITC, CD45 BV510, CD309 AF647, 7AAD, CD144 BV421, and CD31 PE-CY7 (all antibodies from BD Biosciences). Single stains were used for compensation and fluorescence minus one (FMO) was used for setting gates. Antibody-stained cells were assessed using a FACS Canto II (BD Biosciences) flow cytometer, using FACSDiva software (BD Biosciences).

### Immunohistochemistry

Immunohistochemistry was performed on fetal brain tissue sections using standard protocols [45]. Primary antibodies used were mouse monoclonal anti-vascular endothelial growth factor (VEGF) to determine VEGF expression (Novus Biologicals, CO, USA), rabbit polyclonal anti-matrix metalloproteinase-9 (MMP-9) to determine basement membrane breakdown (1:100; Aviva Systems Biology, CA, USA), rabbit polyclonal anti-glucose transporter 1 (GLUT1) to determine endothelial GLUT1 expression (1:200; Abcam, UK), and rabbit polyclonal anti-sheep albumin to determine vascular leakage (1:2000; Accurate Chemical & Scientific, NY, USA). Briefly, sections were dewaxed and rehydrated through graded ethanol immersions. Antigen retrieval was performed by incubating sections with EDTA (pH 9.0) target retrieval solution at 98 °C for 30 min, with Proteinase K (20 µg/ml) at 37C for 30 min before cooling for 20 min at room temperature, or by heating slides immersed in 0.01 M citric acid buffer (pH 6) in a microwave for 4 × 5 min and cooling in the buffer for 20 min. Sections had endogenous peroxidases blocked by incubation with 0.3% H_2_O_2_ in 50% methanol for 15 min. Non-specific binding was blocked by incubating with DAKO serum-free protein block (Agilent Technologies, CA, USA) for 45 min, before sections were incubated overnight with primary antibody at 4 °C. The following day, sections stained using 3,3’-diaminobenzidine (DAB) (MP Biomedicals, OH, USA) were incubated with the appropriate secondary antibody for 45–60 min, then for 45–60 min with streptavidin horseradish peroxidase. Staining was then visualised using DAB, and slides stained for VEGF and MMP-9 counterstained through immersion in 0.5% cresyl-violet for 5 min, as blood vessels that did not express MMP-9 and VEGF were visualised through the counterstain. Coverslips were applied to slides using mounting medium. Negative control sections were included in each run.

Double-label immunofluorescence was used to determine vascular pericyte coverage, using rabbit polyclonal anti-desmin (1:100; Sigma-Aldrich, MO, USA) and mouse monoclonal anti-α-smooth muscle actin (α-SMA) to label the pericyte markers desmin and α-SMA (1:50; Sigma-Aldrich, MO, USA). Similarly, vascular–astrocyte coverage was examined using rabbit polyclonal anti-laminin (1:200; Novus Biologicals, CO, USA) to visualise blood vessels, and mouse monoclonal anti-glial fibrillary acidic protein (GFAP) to visualise astrocytes (1:500; Sigma-Aldrich, MO, USA). Protocols followed those described for single-label immunohistochemistry on the first day, with a step added to block autofluorescence by incubating slides with sodium borohydride (10 mg/ml) in 0.1 M PBS for 3 × 10 min immediately before non-specific blocking was performed. After overnight incubation, slides were incubated for 60–120 min with fluorescence labelled secondary antibodies goat anti-rabbit Alexa 488 (1:1000; Abcam, UK) and goat anti-mouse Alexa 594 (1:1000; Abcam, UK). Slides were then incubated with Hoechst nuclear stain before coverslips were applied using DAKO fluorescence mounting medium (Agilent, CA, USA).

### Image analysis and quantification

Slides stained for VEGF, MMP-9, GLUT1, laminin/GFAP, and desmin/α-SMA were scanned at 40 × magnification to digitalised files using Aperio Scanscope AT Turbo (VEGF, MMP-9, GLUT1; Leica Biosystems, Germany) or Aperio Scanscope FL (laminin/GFAP, desmin/α-SMA; Leica Biosystems, Germany), and viewed with Aperio ImageScope (v12.3.3 for Windows, Leica Biosystems, Germany). Using these files, images were extracted from each of three separate, non-overlapping areas of 500 μm × 500 μm within each of four regions of interest in the brain: the cortical grey mater (Cx), the periventricular white matter (PVWM), the subcortical white matter (sCWM), and the subventricular zone (SVZ). Analysis of these images was performed using Image J software (v2.0.0 for Mac; National Institutes of Health, Bethesda, MD, USA) using previously published techniques [46]. Image analysis and quantification were undertaken by two researchers independently (AB and TY) on coded slides so that all analysis was blinded to the animal group.

#### Vascular density and morphology

Whole cerebral microvessels were imaged on sections stained with laminin/GFAP double-label immunofluorescence by viewing only the green fluorescence channel (*λ* = 488 nm) through which laminin staining could be visualised (Fig. 3). We utilised a macro to determine the following parameters: (i) number of blood vessels present in each field of view; (ii) vascular density—% area occupied by blood vessels per 500μm^2^ field of view; (iii) average size of blood vessels in each field of view. The macro calibrated each section in μm and excluded particles < 10 μm^2^ in total area from analysis to prevent background staining being recognised.

#### VEGF expression

VEGF staining was performed to assess angiogenic signalling. VEGF positive staining was assessed by quantifying the total percentage of each field of view expressing positive staining was measured using an automated macro. A threshold for positive staining was calibrated for each brain region and all images were analysed using this threshold.

#### Basement membrane breakdown

The extent of basement membrane breakdown was quantified by manually counting the number of vessels expressing positive endothelial staining for MMP-9 in each image captured. This number was then used alongside the total number of vessels in each image, identified through cresyl-violet counterstaining, to calculate the percentage of vessels in each 500 μm x 500 μm field of view staining positively for MMP-9.

### Endothelial GLUT1 expression

Microvascular GLUT1 expression provides a marker for metabolic function/dysfunction [47]. A macro was created to identify areas of positive staining. To quantify GLUT1, the vascular wall of cerebral microvessels was isolated as a region of interest (ROI) using a stylus to trace around 1–3 vessels that were selected from each field of view. The % area staining positive for GLUT1 within these isolated regions was then determined based on a staining threshold which was manually set for each image analysed.

#### Co-localisation analysis

To assess vascular pericyte coverage co-localisation of positive staining for desmin and α-SMA, two pericyte markers were undertaken using a double-label immunofluorescence as previously described by our group [14, 15], with slight modification detailed below. The amount of co-localised staining for green (*λ* = 488 nm) and red (*λ* = 594 nm) fluorescence representing desmin and α-SMA was quantified. ROI was obtained using a stylus to isolate the vascular wall of 1–3 vessels per field of view, then determining co-localisation within these isolated regions of interest using Pearson’s correlation coefficient. Pearson’s correlation coefficient uses both signal co-occurrence and correlation to provide a measure of association between two probes [48], making it a useful measure of the degree of co-localisation between two objects labelling the same structure (in this case, desmin and α-SMA).

Co-localisation between the green and red fluorescent staining representing laminin and GFAP was used to quantify astrocyte coverage of vessels using the Manders’ co-localisation coefficient. Manders’ co-localisation coefficient provides a measurement of the co-occurrence between two signals and is thus useful for measuring overlap between two different objects [48]. Automated staining thresholds were set for each image using the Costes method [49], with the M2 Manders’ co-localisation coefficient value providing a representation of the proportion of positive green signal (laminin) overlapping with positive red signal (GFAP) within the entire field of view shown as yellow, and thus the proportion of astrocytic end-feet attachment with vascular tissue.

#### Blood brain barrier integrity

Barrier integrity was assessed through qualitative analysis of albumin extravasation, as we have previously validated for fetal sheep brain Sects. [14, 15] (Fig. 9). Each albumin-stained slide was coded for blinding, then assessed under light microscope (Olympus BX-41, Japan) at 200 × magnification for albumin extravasation. Results were measured on a binary scale determined by the presence or absence of albumin extravasation for each animal, within each region of interest and overall.

### Statistical analysis

Statistical analysis was conducted using Prism 9 (GraphPad Software, CA). Data are presented as mean ± standard error of the mean (SEM). A two-step analysis was used. First, untreated AGA and FGR groups were compared using an unpaired t test. The effects of ECFC administration were then compared via two-way ANOVA with interaction, with fetal growth (AGA vs. FGR) and ECFC treatment (untreated vs. ECFC treatment) as independent variables. Where significant interaction was observed, Tukey’s test was used for post hoc comparisons. Normality of residuals was confirmed for each two-way ANOVA using Kolmogorov–Smirnov test, with failure to pass noted alongside relevant results. Significance for all analyses was set at P ≤ 0.05.

## Results

### Culture and characterisation of ECFCs

**Fig. 1:**
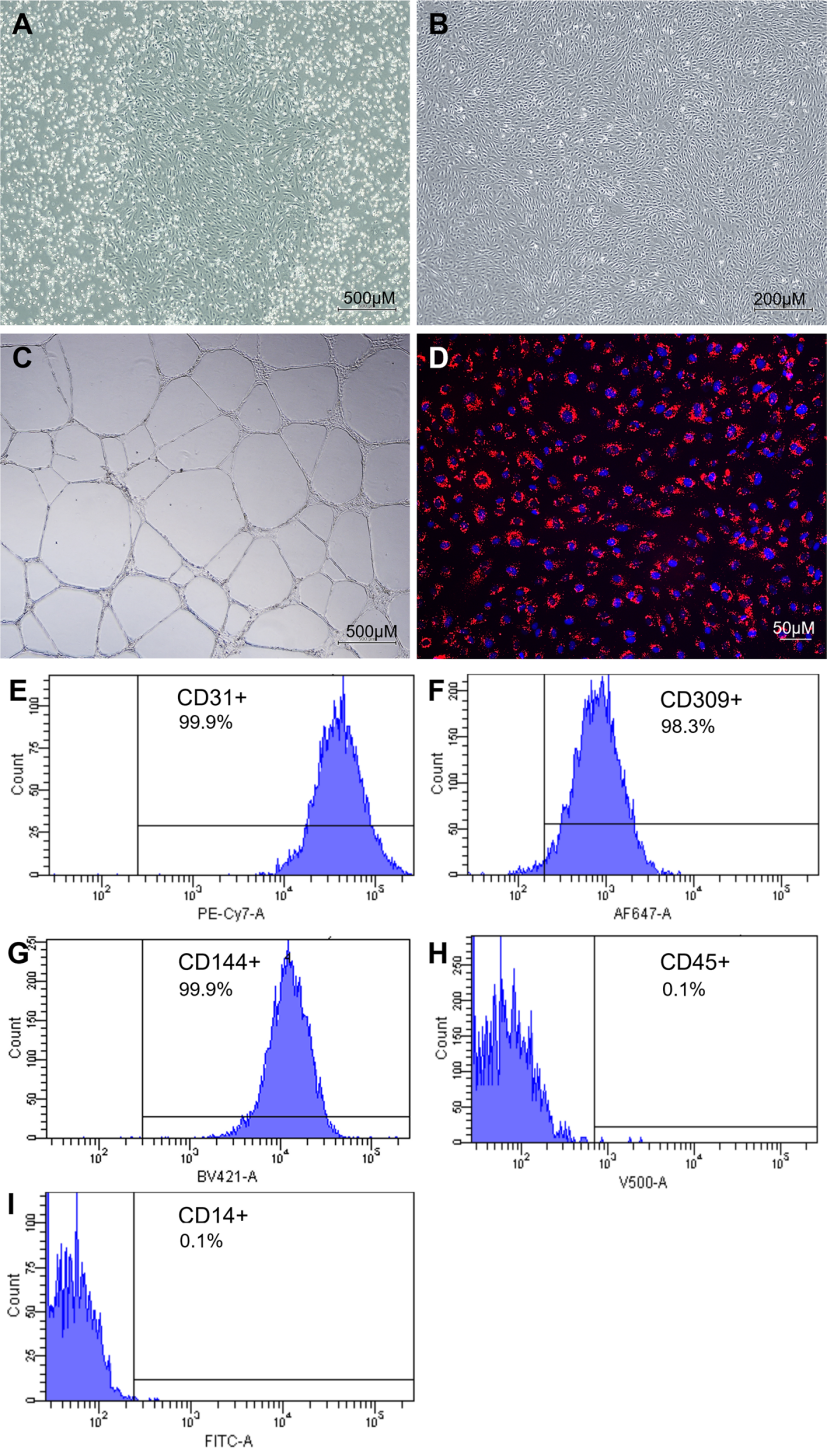
Culture and characterisation of UCB-ECFC. **A** clonal outgrowth of an ECFC colony, **B** expanded ECFCs at passage 3, **C** tube formation assay of ECFCs and **D** acLDL uptake by cultured ECFCs, acLDL (red) and nuclei stained with Hoechst (blue). Flow cytometric analysis for common ECFC surface markers; **E** CD31, **F** CD309, **G** CD144, **H** CD45 and **I** CD14

ECFCs were cultured from UCB from term healthy mothers. Adherent colonies were observed between days 7–18 of culture displaying cobblestone morphology and subcultured. ECFCs appeared as highly proliferative cobblestone cells (Fig. 1A, B). To assess ECFC functionality post-culture, a tube formation assay and acLDL uptake assay were performed. When cultured on Matrigel, ECFCs were shown to form connecting capillary-like structures within 24 h of culture (Fig. 1C). ECFCs also showed positive uptake of acLDL-Dil (Fig. 1D) confirming the cells showed the functional characteristics associated with ECFCs.

Flow cytometry was used to assess the surface marker expression of the ECFCs to confirm the cells express typical ECFC markers, CD31, CD309, and CD144 as well as showing negative expression of the common leukocyte marker CD45 and monocyte marker CD14. The cultured ECFCs showed 99.9% positivity for CD31 (Fig. 1E), 98.3% positivity for CD309 (Fig. 1F), 99.9% positivity for CD144 (Fig. 1G), while showing negative 0.1% for CD45 (Fig. 1H) and CD14 (Fig. 1I). These studies confirm the cultured ECFCs show phenotype and functionality of ECFCs of endothelial origin.

### Animal characteristics

**Fig. 2:**
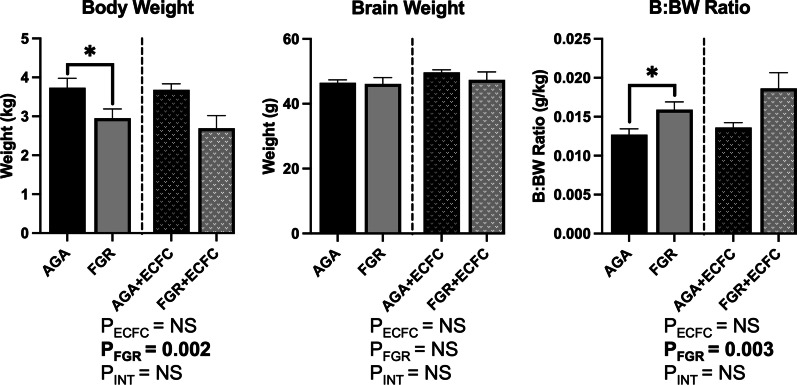
Data are mean ± SEM for body weight, brain weight, and brain/body weight (B/BW) ratios in AGA (Black; n = 7), FGR (Grey; n = 5), AGA + ECFC (Black patterned; n = 6), and FGR + ECFC (Grey patterned; n = 6). Data were examined using a two-step analysis, with AGA and FGR compared first via unpaired t test, and then, all groups compared using two-way ANOVA with FGR and ECFC treatment as variables. *P ≤ 0.05 using unpaired t test for AGA versus FGR. Two-way ANOVA results for main effects (P_ECFC_, P_FGR_) and interaction (P_INT_) for each brain region are presented below the relevant graph (NS = P > 0.05)

A total of 24 fetal sheep (17 M, 7 F) were included in analyses, arranged in four groups: AGA (n = 7), FGR (n = 5), AGA + ECFC (n = 6), and FGR + ECFC (n = 6). Biometric characteristics of animals in each group are presented in Fig. 2. The untreated FGR fetuses weighed significantly less than the untreated AGA animals (P = 0.05), and brain/body weight ratio was increased (P = 0.03) but there was no difference in brain weights (P = 0.84). Two-way ANOVA found FGR significantly reduced body weight (ANOVA, main effect for AGA vs. FGR, P = 0.002) and significantly increased brain/body weight (B/BW) ratio (ANOVA, main effect for AGA vs. FGR, P = 0.003), but had no significant effect on brain weight. ECFC treatment did not have a significant effect on body weight, brain weight, or B/BW ratio.

### Vascular structure

We assessed whether exogenous ECFCs could be detected within the fetal brain by examining whether the fluorescently labelled ECFCs could be visualised under the fluorescence microscope (Olympus BX-41, Japan). Fluorescently labelled ECFCs were not observed in any brain sections, with no identifiable differences noted between slides from animals in groups that had received ECFC treatment, and those that had not received ECFC treatment.

**Fig. 3:**
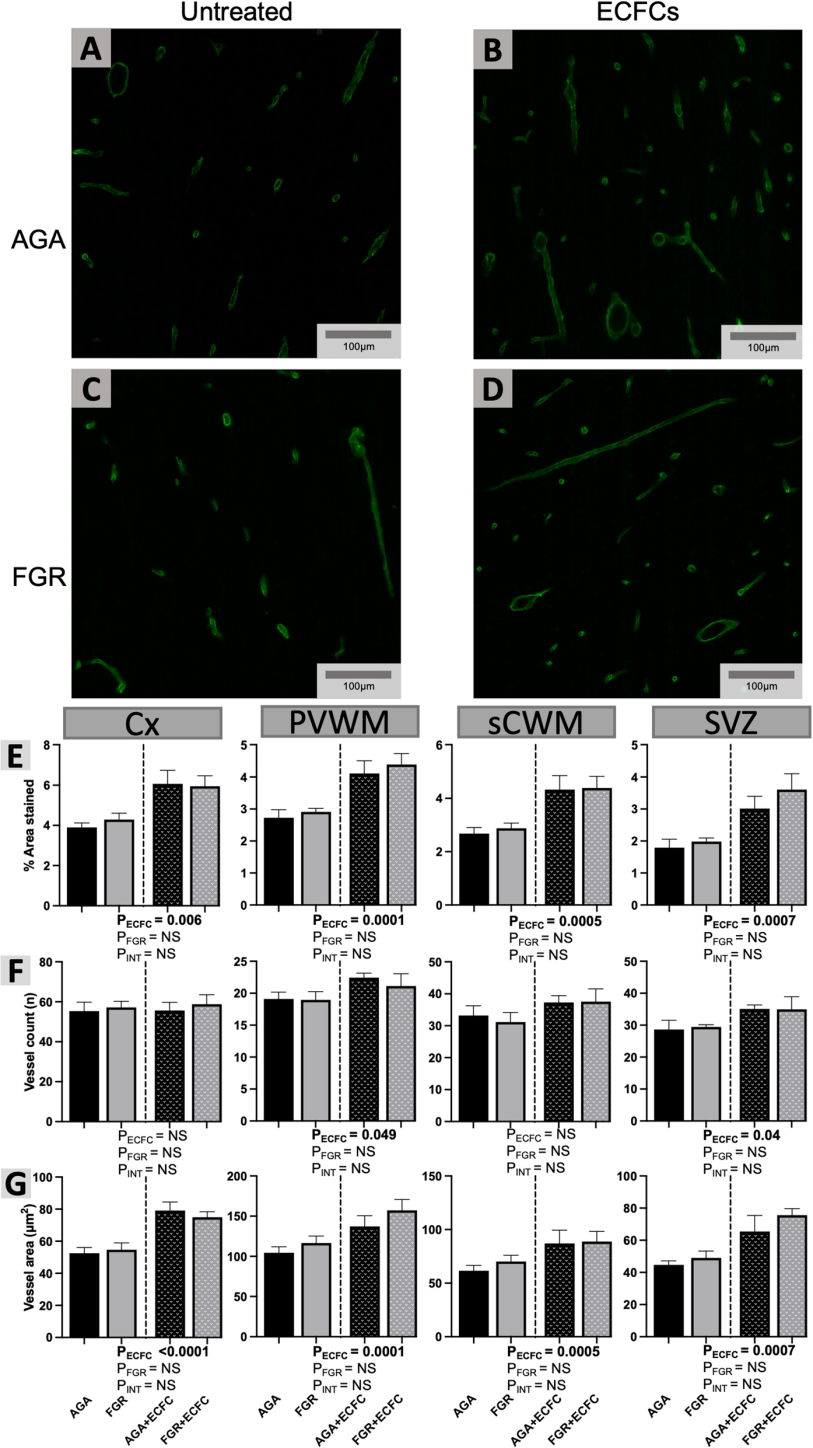
**A-D** Representative photomicrographs of laminin immunofluorescence staining in the sCWM. **E–G.** Data are mean ± SEM for (**E**) average % of each field of view displaying positive laminin staining, (**F**) average number of blood vessels identified per field of view, and (**G**), average size of each blood vessel staining positively for laminin (μm^2^) as calculated in the Cx, PVWM, sCWM, and SVZ from AGA (Black; n = 7), FGR (Grey; n = 5), AGA + ECFC (Black patterned; n = 6), and FGR + ECFC (Grey patterned; n = 6). Data were examined using a two-step analysis, with AGA and FGR compared first via unpaired t test, then all groups compared using two-way ANOVA with FGR and ECFC treatment as variables. Two-way ANOVA results for main effects (P_ECFC_, P_FGR_) and interaction (P_INT_) for each brain region are presented below the relevant graph (NS = P > 0.05)

Representative images for laminin staining and analysis are presented in Fig. 3. There were no significant differences in vessel density, number, or size between untreated AGA and FGR groups (P > 0.05). Two-way ANOVA showed that ECFC treatment increased vascular density in the Cx (ANOVA, main effect for untreated vs. ECFC, P = 0.0006), PVWM (ANOVA, main effect for untreated vs. ECFC, P = 0.0001), sCWM (ANOVA, main effect for untreated vs. ECFC, P = 0.0005), and SVZ (ANOVA, main effect for untreated vs. ECFC, P = 0.0007). Using mean values from all untreated animals together, and all ECFC-treated animals together, vascular density was increased in ECFC-treated foetuses by 48% in the Cx (Untreated: *x*ˉ = 4.0 ± 0.2; ECFC: *x*ˉ = 6.0 ± 0.4), 52% in the PVWM (Untreated: *x*ˉ = 2.8 ± 0.1; ECFC: *x*ˉ = 4.2 ± 0.3), 58% in the sCWM (Untreated: *x*ˉ = 2.8 ± 0.2; ECFC: *x*ˉ = 4.4 ± 0.3), and 77% in the SVZ (Untreated: *x*ˉ = 1.9 ± 0.2; ECFC: *x*ˉ = 3.3 ± 0.3).

ECFC treatment induced a significant increase in the average number of vessels per field of view in the PVWM (ANOVA, main effect for untreated vs. ECFC, P = 0.049) and SVZ (ANOVA, main effect for untreated vs. ECFC, P = 0.04). The number of vessels was increased in ECFC-treated fetuses by 14% in the PVWM (Untreated: *x*ˉ = 19.0 ± 0.8; ECFC: *x*ˉ = 21.8 ± 1.0), and by 20% in the SVZ (Untreated: *x*ˉ = 29.0 ± 1.7; ECFC: *x*ˉ = 35.0 ± 2.0). Vessel size was significantly increased with ECFC treatment in the Cx (ANOVA, main effect for untreated vs. ECFC, P < 0.0001), PVWM (ANOVA, main effect for untreated vs. ECFC, P = 0.004), sCWM (ANOVA, main effect for untreated vs. ECFC, P = 0.02), and SVZ (ANOVA, main effect for untreated vs. ECFC, P = 0.0007). Average vessel sizes were increased in ECFC-treated fetuses by 44% in the Cx (Untreated: *x*ˉ = 53.5 ± 2.6; ECFC: *x*ˉ = 77.0 ± 3.1), 35% in the PVWM (Untreated: *x*ˉ = 109.5 ± 5.7; ECFC: *x*ˉ = 147.3 ± 9.5), 35% in the sCWM (Untreated: *x*ˉ = 65.1 ± 3.9; ECFC: *x*ˉ = 87.9 ± 7.4), and by 52% in the SVZ (Untreated: *x*ˉ = 46.5 ± 2.3; ECFC: *x*ˉ = 70.6 ± 5.3).

**Fig. 4:**
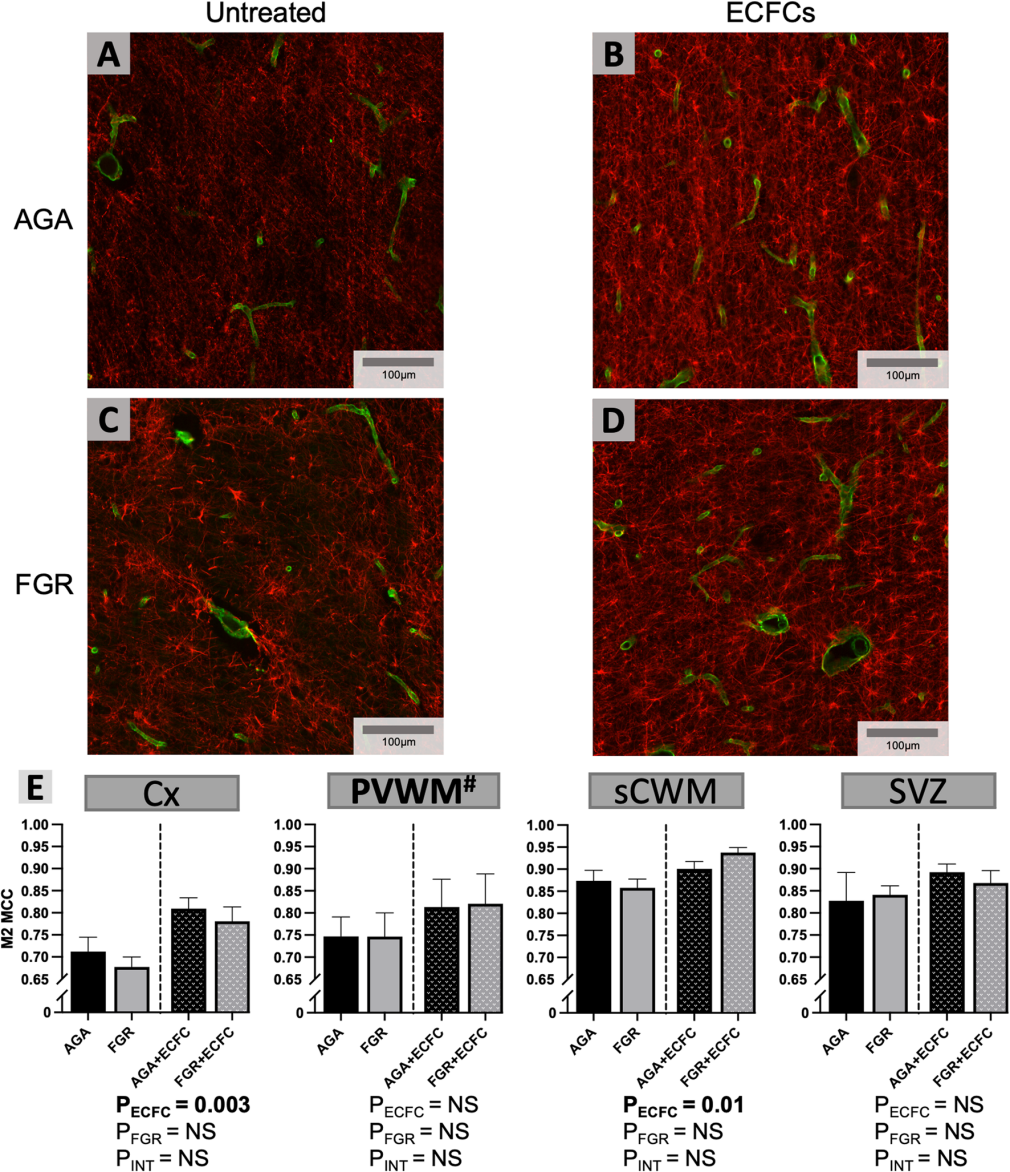
**A-D** Representative photomicrographs of double-label laminin (green) and GFAP (red) immunofluorescence staining in the sCWM. **E** Data are mean ± SEM average M2 Manders’ co-localisation coefficient (MCC) per field of view as calculated in the Cx, PVWM, sCWM, and SVZ from AGA (Black; n = 7), FGR (Grey; n = 5), AGA + ECFC (Black patterned; n = 6), and FGR + ECFC (Grey patterned; n = 6). Data were examined using a two-step analysis, with AGA and FGR compared first via unpaired t test, and then, all groups were compared using two-way ANOVA with FGR and ECFC treatment as variables. Two-way ANOVA results for main effects (P_ECFC_, P_FGR_) and interaction (P_INT_) for each brain region are presented below the relevant graph (NS = P > 0.05). ^#^P < 0.05 for Kolmogorov–Smirnov test of residuals in two-way ANOVA analysis of PVWM

Figure 4 provides representative examples of laminin/GFAP double-label staining to assess vascular–astrocyte coverage. Significant differences were not found between untreated AGA and FGR groups (P > 0.05). Increased vascular–astrocyte coverage was observed following ECFC treatment in the Cx (ANOVA, main effect for untreated vs. ECFC, P = 0.003), and the sCWM (ANOVA, main effect for untreated vs. ECFC, P = 0.01).

**Fig. 5:**
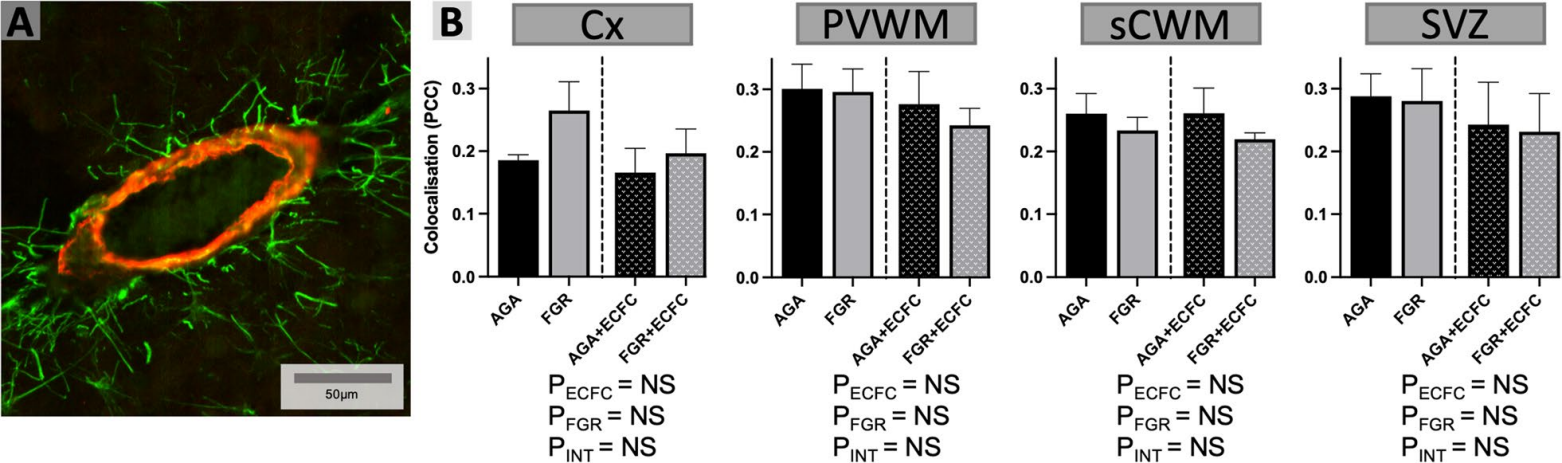
**A** Representative photomicrograph of double-label desmin (green) and αSMA (red) immunofluorescence staining in the sCWM. **B** Data are mean ± SEM average Pearson’s correlation coefficient (PCC) for vessels analysed in the Cx, PVWM, sCWM, and SVZ from AGA (Black; n = 7), FGR (Grey; n = 5), AGA + ECFC (Black patterned; n = 6), and FGR + ECFC (Grey patterned; n = 6). Data were examined using a two-step analysis, with AGA and FGR compared first via unpaired t test, and then, all groups were compared using two-way ANOVA with FGR and ECFC treatment as variables. Two-way ANOVA results for main effects (P_ECFC_, P_FGR_) and interaction (P_INT_) for each brain region are presented below the relevant graph (NS = P > 0.05)

The extent of co-localisation between fluorescently labelled proteins desmin and α SMA was used to determine the density of vascular pericyte coverage (Fig. 5). We found no significant differences between untreated AGA and FGR groups, and no significant effect on vascular pericyte coverage due to ECFC treatment.

**Fig. 6:**
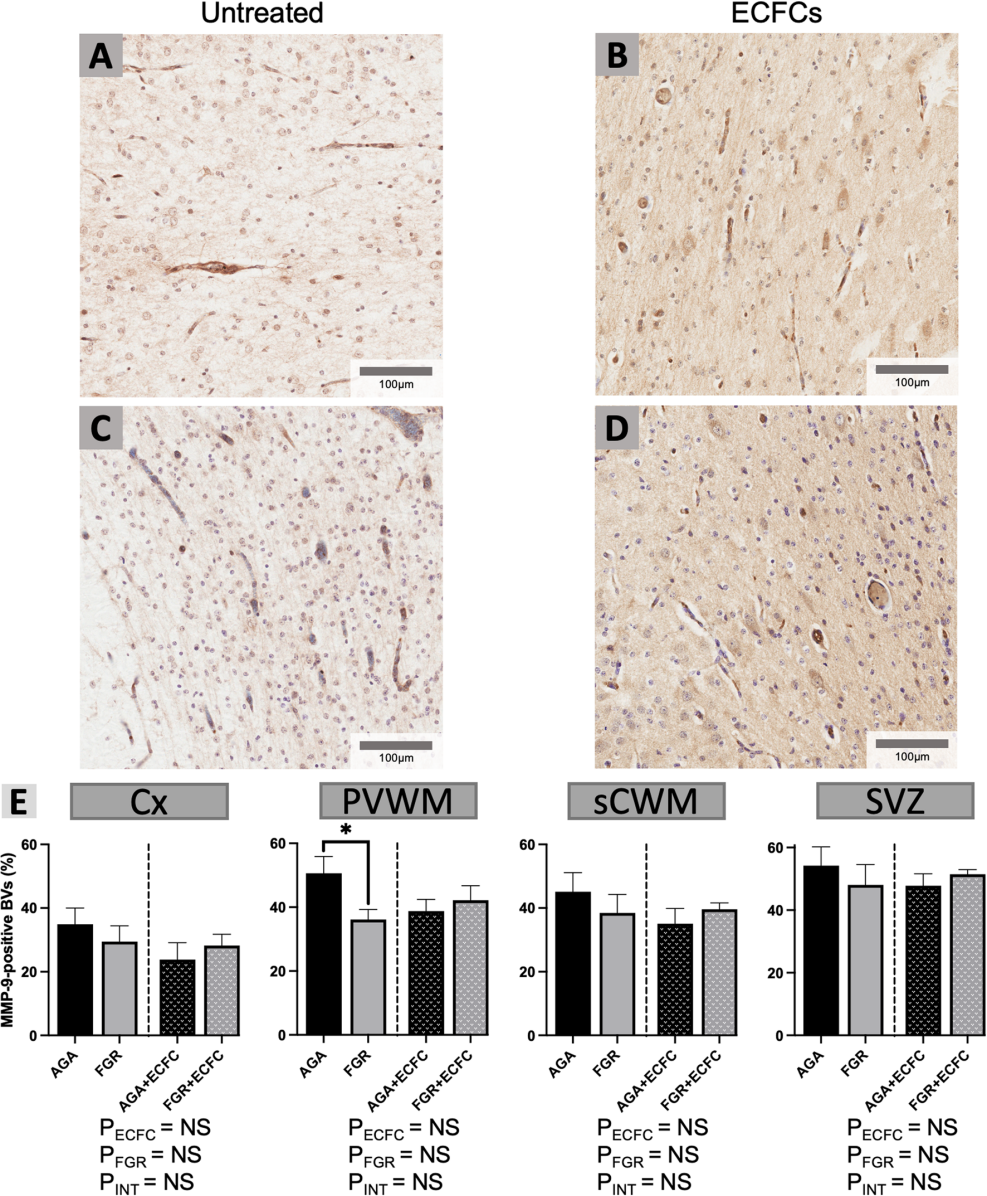
**A–D** Representative photomicrographs of MMP-9 immunohistochemical staining in the sCWM. **E** Data are mean ± SEM for % of identified blood vessels staining positively for MMP-9 expression as calculated in the Cx, PVWM, sCWM, and SVZ from AGA (Black; n = 7), FGR (Grey; n = 5), AGA + ECFC (Black patterned; n = 6), and FGR + ECFC (Grey patterned; n = 6). Data were examined using a two-step analysis, with AGA and FGR compared first via unpaired t test, then all groups compared using two-way ANOVA with FGR and ECFC treatment as variables. *P ≤ 0.05 using unpaired t test for AGA versus FGR. Two-way ANOVA results for main effects (P_ECFC_, P_FGR_) and interaction (P_INT_) for each brain region are presented below the relevant graph (NS = P > 0.05)

The matrix metalloproteinases are a family of endopeptidases that are capable of degrading vascular extracellular matrix and are therefore implicated in BBB breakdown. Here, we assessed whether MMP-9 expression was altered by either FGR or ECFC administration. The percentage of vessels in each field of view manually assessed as expressing positive staining for MMP-9 was used to determine overall vascular MMP-9 activity in each brain region analysed. Representative examples of this staining are presented alongside analysis in Fig. 6. FGR was associated with a significant reduction in MMP-9 activity within the PVWM in untreated FGR groups, compared with untreated AGA groups (P = 0.05), while ECFC treatment did not significantly alter vascular MMP-9 activity in any of the brain regions analysed.

### Vascular function

**Fig. 7:**
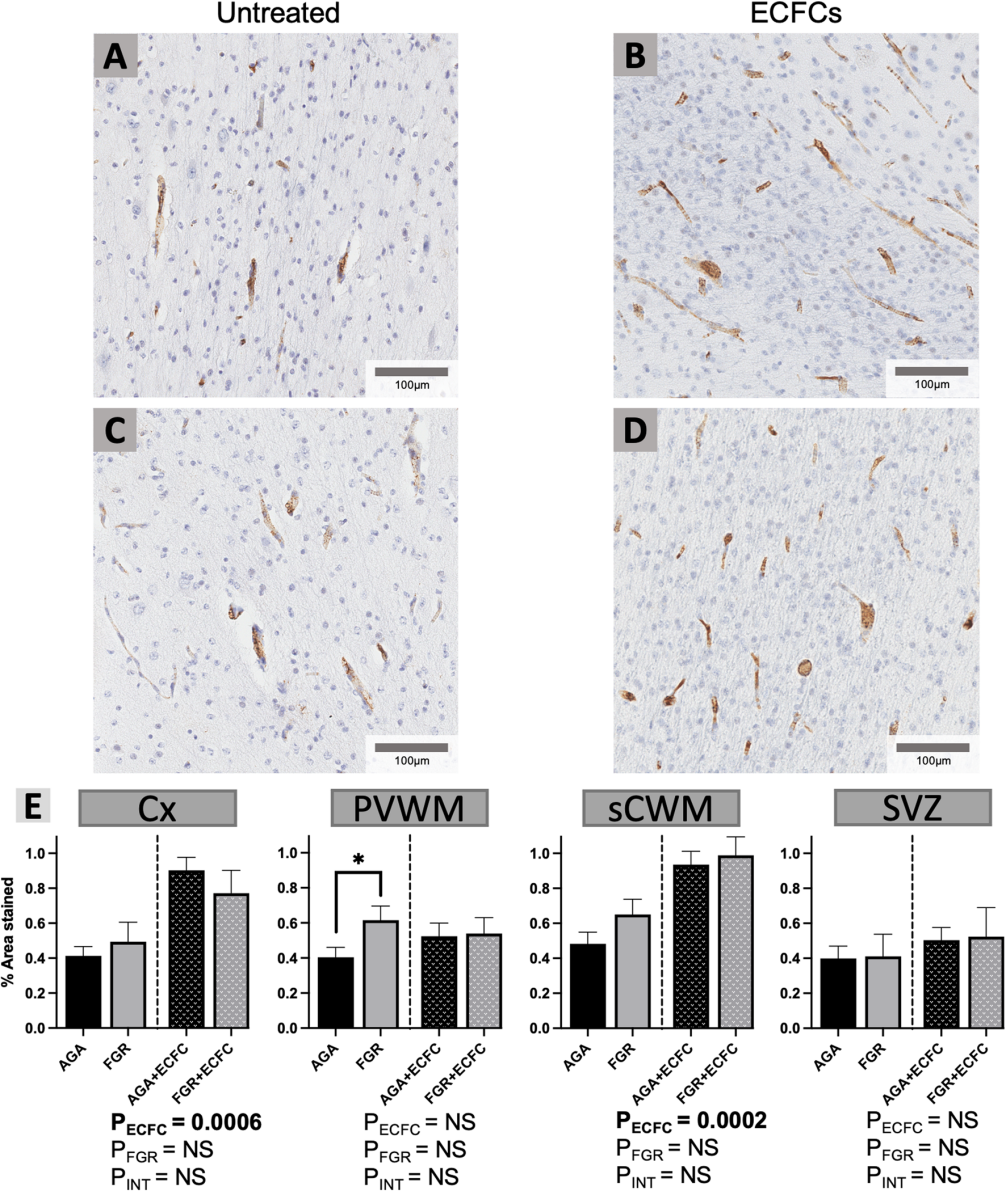
**A–D** Representative photomicrographs of VEGF immunohistochemical staining in the sCWM. **E** Data are mean ± SEM for % of each field of view displaying positive VEGF staining as calculated in the Cx, PVWM, sCWM, and SVZ from AGA (Black; n = 7), FGR (Grey; n = 5), AGA + ECFC (Black patterned; n = 6), and FGR + ECFC (Grey patterned; n = 6). Data were examined using a two-step analysis, with AGA and FGR compared first via unpaired t test, then all groups compared using two-way ANOVA with FGR and ECFC treatment as variables. *P ≤ 0.05 using unpaired t test for AGA vs FGR. Two-way ANOVA results for main effects (P_ECFC_, P_FGR_) and interaction (P_INT_) for each brain region are presented below the relevant graph (NS = P > 0.05)

FGR was associated with significantly increased VEGF expression within the PVWM in untreated FGR groups, compared with untreated AGA groups (P = 0.05). Increased VEGF expression was also found following ECFC treatment in the Cx (ANOVA, main effect for untreated vs. ECFC, P = 0.0006) and the sCWM (ANOVA, main effect for untreated vs. ECFC, P = 0.0002), with representative images provided in Fig. 7.

**Fig. 8:**
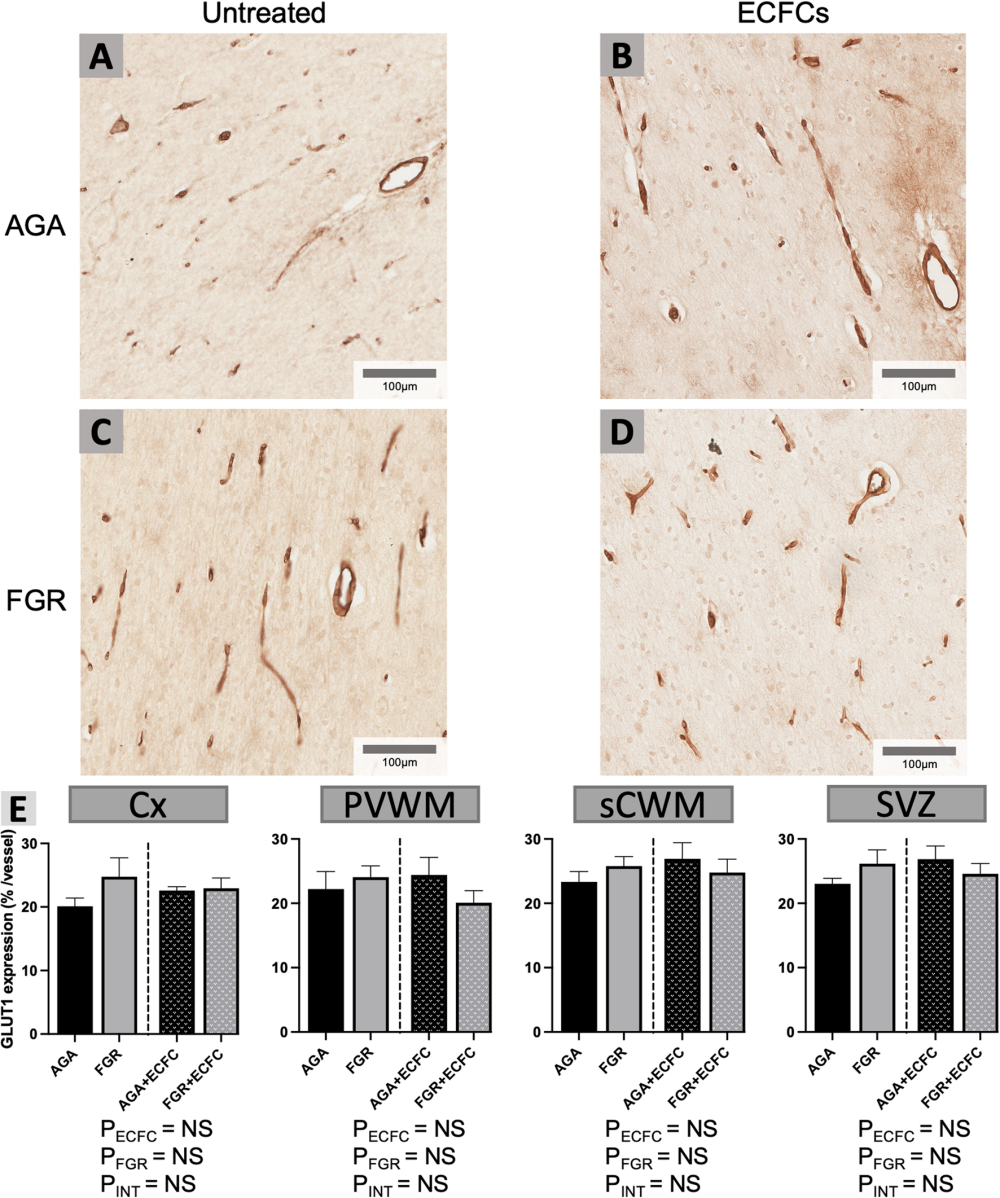
**A–D** Representative photomicrographs of GLUT1 immunohistochemical staining in the sCWM. **E** Data are mean ± SEM for % of each vessel wall analysed displaying positive GLUT1 staining in each group. Data presented as mean ± SEM. as calculated in the Cx, PVWM, sCWM, and SVZ from AGA (Black; n = 7), FGR (Grey; n = 5), AGA + ECFC (Black patterned; n = 6), and FGR + ECFC (Grey patterned; n = 6). Data were examined using a two-step analysis, with AGA and FGR compared first via unpaired t test, then all groups compared using two-way ANOVA with FGR and ECFC treatment as variables. Two-way ANOVA results for main effects (P_ECFC_, P_FGR_) and interaction (P_INT_) for each brain region are presented below the relevant graph (NS = P > 0.05)

Vascular GLUT1 expression provides an insight into the metabolic function and activity of blood vessels [47]. The proportion of the vascular wall staining positively for GLUT1 was used to determine GLUT1 expression, with analysis of this staining presented alongside representative examples in Fig. 8. No significant differences were found between untreated AGA and FGR groups, and ECFC treatment did not have a significant effect on endothelial GLUT1 expression in any of the brain regions analysed.

**Fig. 9:**
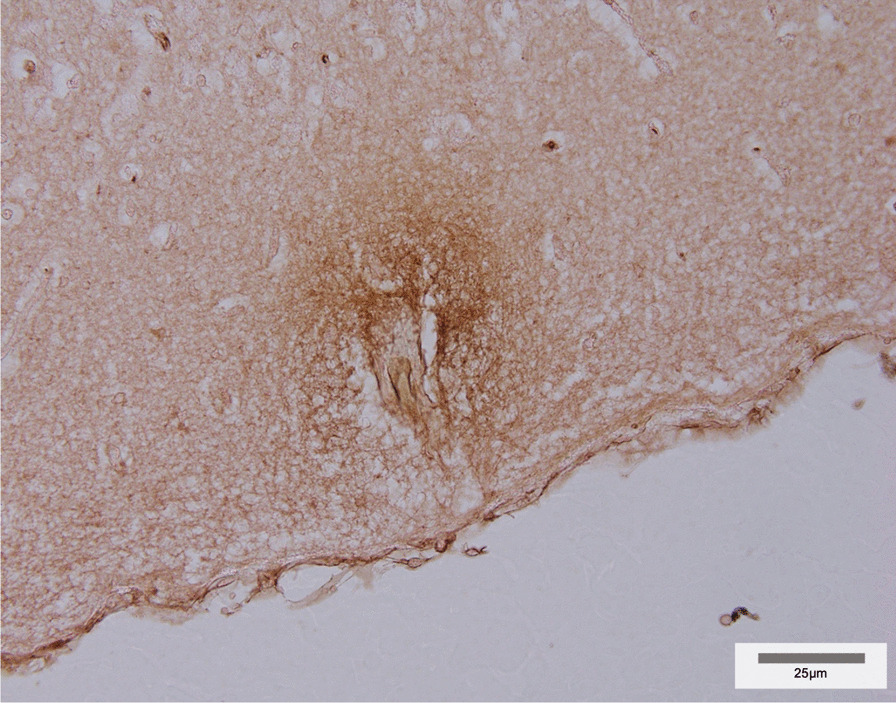
Example photomicrograph at 400 × magnification depicting albumin extravasation into the brain parenchyma in the cortex

Barrier integrity was investigated by assessing albumin extravasation as seen in a representative image in Fig. 9, with stained slides evaluated semi-quantitatively and presented in Table 1. Albumin extravasation was present in 4/5 untreated FGR animals, including within white matter regions of two of these four. Following ECFC treatment, albumin extravasation was found in only 3/5 FGR animals, and in white matter regions of just one.

**Table 1:** Results of staining for albumin extravasation in each group

		Cx	PVWM	sCWM	SVZ	Total
Untreated	**AGA**	2/7	0/7	0/7	0/7	2/7
	**FGR**	3/5	0/5	2/5	2/5	4/5
**ECFC**	**AGA**	2/6	0/6	0/6	0/6	2/6
	**FGR**	2/5	1/5	1/5	0/5	3/5

Numbers represent the number of animals in which albumin extravasation was observed within each brain region of interest, as well as within the brain as a whole (total). Results are presented out of the total number of animals analysed within each group.

## Discussion

We provide the first evidence that human UCB-derived ECFCs promote cerebral angiogenesis and enhance NVU development in preterm fetal sheep. Specifically, our results demonstrate that human UCB-derived ECFCs increase vascular density and angiogenic signalling, and enhance interactions between cerebral vessels and their supporting NVU component astroglial cells. These results demonstrate that ECFCs may provide a useful therapeutic strategy to protect against perinatal brain injury that is moderated by developmental deficits in the NVU.

In the current study, we showed that we could successfully expand clonal endothelial progenitor cells that expressed the characteristic endothelial surface makers CD31, CD144, and CD309 while lacking expression of the monocyte marker CD14 and common leukocyte marker CD45, supporting that these cells isolated from UCB are of endothelial origin and not haematopoietic [44, 50]. These expanded cells also showed in vitro functionality common with ECs, such as forming capillary-like structures when cultured on a basement membrane and the ability to bind acetylated LDL [44]. This indicates that as well as expressing appropriate surface markers for ECFCs these cells retained their functionality post-expansion.

ECFC administration increased vascular density in both appropriately grown and FGR fetuses. This effect was seen in all brain regions analysed, though the impact of ECFC administration appeared greater in FGR animals within the PVWM and SVZ. This supports our previous findings highlighting these regions as vulnerable to reduced vascular density associated with FGR [14]. We think the cerebral white matter is extremely vulnerable to perfusion-related injury because it receives only 25% of the blood flow of cortical grey matter and has a maturation impairment in ability to autoregulate blood flow [51]. Our results demonstrate that while ECFC treatment induced a region-specific increase in the number of vessels, the increased vascular density throughout the brain was primarily attributable to an increase in the overall size of blood vessels. Cerebral blood vessel number was significantly increased in both the PVWM and SVZ, while the increase in vessel size was proportionally larger and observed across all brain regions analysed. These outcomes support results from a previous study in newborn piglets, which found that treatment with a combination of ECFCs and mesenchymal stromal cells (MSC) promoted an increase in the density and length of cortical vessels, where MSCs alone did not [52]. Interestingly however, in the piglet study it was observed that increased vascular density only appeared to correct the deficiency that was apparent in FGR offspring, with no effect observed when the same treatment was administered to appropriately grown piglets. Similarly, UCB mononuclear cells (which include ECFCs plus other cell types [30, 53, 54]) demonstrate a capacity to upregulate proliferation in ovine NVU vessels, but this effect is only apparent in brains affected by FGR [15]. It was surprising that we did not detect obvious deficits in the cerebral vasculature of FGR brains in the current study, where previously our group has shown reductions in vascular density and endothelial proliferation [14]. The key difference in these studies is the age of the animals at post-mortem, with previous differences seen in one-day-old newborns delivered at term. In our study, we did, however, observe an angiogenic effect of ECFCs that was above and beyond normal levels observed in the appropriately grown brain. The consequences for an over-supply of blood vessels are not known. Together, these data suggest that a targeted treatment approach using ECFCs in situations of neurovascular dysfunction would be recommended.

In response to ECFC treatment, the increase in vascular density was accompanied by increased astrocyte association with blood vessels, and increased VEGF expression; these are critical components for vasculogenesis. Astrocyte function and vascular interactions are critical for vessel growth and integrity, with a paucity of astrocytes implicated in blood vessel fragility in the developing brain [55]. Our results demonstrate increased vascular coverage by astrocyte end-feet in response to ECFC treatment. Importantly, this pattern resembled that of vascular density, with astrocytic end-feet coverage of vessels increased above baseline levels following treatment across both FGR and appropriately grown brains. This corresponding increase in both vascular coverage and astrocytic end-feet is unsurprising, given the interdependent relationship between astrocytes and vessels as components of the developing NVU. Astrocytes play an essential role in neurovascular development through their participation in both spatial and temporal guiding of angiogenic pathways through neural tissue [56, 57], and close associations with vascular endothelial cells are crucial to their differentiation from earlier precursors [58, 59]. Underlying the interconnected nature of NVU components further, the increased VEGF expression that we observed is likely a result of vascular–astrocyte interactions. Astrocyte-derived VEGF plays a key role in promoting growth and stability of developing vessels, particularly in response to hypoxic conditions [60–63]. Alternatively, enhanced astrocytic vascular coverage may occur secondary to an upregulation of VEGF expression. VEGF is closely associated with astrocyte proliferation [64] and is postulated to promote astrocyte growth and differentiation [65], thus changes to astrocyte activity and vascular interaction may be a response to increased VEGF produced by ECFCs [66, 67]. We observed a strong region-specific response within the brain following ECFC administration, with vascular density, astrocyte attachment and VEGF all increased in the cortex and the subcortical white matter. The particular response of these regions to treatment may reflect the considerable development they undergo around the gestational age investigated, with cortical volume and surface area increasing dramatically between 28 and 40 weeks in humans [68].

Though our results support the potential of exogenous ECFC administration to improve NVU development, the absence of substantial differences in NVU characteristics associated specifically with FGR was interesting but unexpected. FGR was associated only with significantly increased VEGF expression and reduced MMP-9 activity in the PVWM. A lack of FGR-associated changes to the NVU is at odds with previous preclinical evidence. We have previously reviewed this literature, which demonstrates the adverse impacts of pregnancy complications, including FGR, on NVU component cells and function [6]. Most relevant to our investigation are studies demonstrating associations between FGR and changes in astrocyte density [69, 70], vessel proliferation [14, 71], and vascular pericyte coverage [14, 15]. A previous study by Castillo-Melendez et al. showed that FGR was associated with a reduction in vascular density and number, endothelial proliferation, vascular–astrocyte and pericyte coverage, and barrier integrity [14]. The Castillo-Melendez study induced placental insufficiency using the same technique as used here, although the previous study induced late-onset FGR while here we employed early-onset, and post-mortem tissue collection was previously performed in one-day-old lambs that were approximately 20 days older than the fetuses in this current study. It is therefore possible that structural and functional deficits in the NVU predominantly arise in late gestation, when we would expect the degree of fetal hypoxaemia to be at its greatest. Vascular proliferation is increased in late gestation, particularly in the cortex [72], and this enhanced angiogenic activity may also contribute to the development of NVU deficits during this period. The potential for enhanced NVU vulnerability to FGR in late gestation is supported by Chand et al., who investigated FGR-associated NVU alterations in newborn piglets at 4 days of age. They found the condition was linked to reductions in vessel density and length, astrocyte maturity, and vascular end-feet coverage, as well as vascular barrier function, all of which were improved following combination ECFC and MSC treatment [52]. Other pregnancy complications may also impact development of the NVU, with a recent study showing that cells of the NVU are adversely affected by chronic inflammation, with prolonged lipopolysaccharide exposure resulting in reduced cerebral vascular density and glial coverage [73]. The apparent discordance we have highlighted between results of the present investigation and findings of other studies highlights the relative lack of understanding that exists regarding the developing NVU, and the importance of continued efforts to develop this knowledge.

Although we were principally interested in cerebral vasculature and the NVU, FGR is also associated with altered microvascular density across the kidneys, retina, and musculature, among other systems [74]. These widespread vascular deficits may arise as a result of EPC dysfunction also associated with FGR. Circulating maternal EPCs increase in pregnancy with increase in gestational age [75, 76]; however in FGR pregnancies, circulating EPC numbers are substantially lower than in healthy equivalents [77]. Reduced EPC counts in FGR extend to the UCB, where EPCs also display increased senescence alongside poorer vasculogenic function than those of healthy pregnancies [38, 39, 78], with these impairments in EPC numbers and functioning extending beyond the perinatal period into later life [79]. In light of the well-established EPC dysfunction in FGR, it is likely that these cells have a role to play in NVU alterations associated with the condition. This hypothesis is supported by the results of the present investigation, which demonstrate ECFC administration leading to enhancements in NVU domains that have previously shown evidence of impaired development in the setting of FGR. The improvements we have observed following treatment also support the potential for the development of ECFC-based therapies to substantially enhance NVU outcomes in FGR individuals.

The response of the developing NVU to treatment was encouraging for the prospects of ECFC-based therapies to ameliorate dysfunction associated with FGR; however, it is notable that this response also extended to appropriately grown brains. Though the proangiogenic potential of ECFCs means this observation is somewhat unsurprising, it does raise questions regarding the consequences of enhanced vascular proliferation in the otherwise healthy brain. Vascular networks that are established at an increased rate may be more fragile, or vulnerable to dysregulation [80]. Conversely, ECFC administration mediates functional enhancement and reparative effects at the BBB [81–83], indicative of increased angiogenesis following ECFC treatment that may enhance vascular stability. This is supported by our observations of albumin extravasation which provides an assessment of BBB stability, with our results showing that ECFC administration may reduce BBB ‘leak’ in the subcortical white matter and subventricular zone, although this finding requires substantiation in a more robust experimental model of NVU dysfunction.

Though the consequences of enhanced vascular proliferation in the healthy brain may provide important insights into the mechanisms and safety of ECFC administration, their direct relevance is limited. ECFC-based therapies are targeted towards conditions of vascular compromise and as such are unlikely to be administered to healthy infants. Similarly, practical realities and safety considerations mean such therapies are unlikely to be administered directly to the fetus in utero, as was performed in this investigation, and we acknowledge this as a limitation of this work. With the primary purpose of this investigation being to determine whether ECFC administration could enhance NVU development, treatment was delivered in utero as this provided the best method for maintaining preterm animals chronically, without the confounding effects of birth and neonatal care. This was a proof-of-concept study, and an appropriate next step would be to deliver FGR lambs preterm and commence respiratory support as would occur clinically [15] and then administer ECFCs via an intranasal route [84, 85]. The 127-day gestational age of fetal lambs analysed in this investigation corresponds to the late preterm period of human gestation [86], during which FGR is associated with vulnerability to certain neurovascular events [6]; however more commonly with early-onset FGR, the infant is born preterm. Future investigations should consider how the NVU response to ECFC administration is altered by factors such as whether treatment is given pre-or postnatally, as well as the age of the recipient at administration.

In summary, we have presented findings to validate and assess the potential of ECFCs as a therapeutic agent for treatment of disruption of the NVU in our fetal sheep model of FGR. This work was proof-of-concept as it is not envisaged that ECFCs would be administered to the fetuses in a clinical setting. However, our results demonstrate that administration of exogenous ECFCs enhances perinatal NVU development, leading to increased vascular size and improved structural integrity that is likely to be mediated, at least in part, through tight astrocytic end-feet attachment with the cerebral vasculature. These effects may augment cerebrovascular adaptations to FGR and enhance oxygen and nutrient delivery to the developing brain. Our results indicate that ECFCs may be an effective therapeutic strategy to target deficits in the NVU, in infants affected by FGR or with other NVU deficiencies such as those that occur with preterm birth and intrauterine inflammation [6]. ECFCs can be obtained from multiple sources and here we used umbilical cord blood-derived ECFCs, highlighting the potential for development of allogeneic ECFC-based treatments. Further ongoing preclinical investigations should consider the effect of neonatal ECFC administration on long-term structural and functional outcomes associated with FGR and other conditions of perinatal compromise in which the cerebral vasculature and NVU cells demonstrate altered development.

## Conclusions

ECFC administration enhanced development of NVU components in both the AGA and FGR fetal brain. Further investigation is required to assess how to optimise the enhanced angiogenic capabilities of ECFCs to provide a therapeutic strategy to protect the developing NVU against vulnerabilities associated with FGR.

## Data Availability

The data that support the findings of this study are available on reasonable request from the corresponding author.

## Abbreviations

acLDL: Acetylated low-density lipoprotein
AGA: Appropriate for gestational age
BBB: Blood brain barrier
Cx: Cortex
ECFC: Endothelial colony forming cell
EPC: Endothelial progenitor cell
FBS: Fetal bovine serum
FGR: Fetal growth restriction
GFAP: Glial fibrillary acidic protein
GLUT1: Glucose transporter 1
MMP-9: Matrix metalloproteinase-9
MNC: Mononuclear cells
MSC: Mesenchymal stromal cell
NVU: Neurovascular unit
PBS: Phosphate buffered saline
PVWM: Periventricular white matter
ROI: Region of interest
sCWM: Subcortical white matter
SVZ: Subventricular zone
UCB: Umbilical cord blood
VEGF: Vascular endothelial growth factor
α-SMA: α-Smooth muscle actin
